# Comparison of the composition of lymphocyte subpopulations in non-relapse and relapse patients with squamous cell carcinoma of the head and neck before, during radiochemotherapy and in the follow-up period: a multicenter prospective study of the German Cancer Consortium Radiation Oncology Group (DKTK-ROG)

**DOI:** 10.1186/s13014-021-01868-5

**Published:** 2021-07-31

**Authors:** Minli Niu, Stephanie E. Combs, Annett Linge, Mechthild Krause, Michael Baumann, Fabian Lohaus, Nadja Ebert, Ingeborg Tinhofer, Volker Budach, Jens von der Grün, Franz Rödel, Anca-Ligia Grosu, Gabriele Multhoff

**Affiliations:** 1 Center for Translational Cancer Research (TranslaTUM), Radiation Immuno-Oncology Group, Klinikum rechts der isar, TU München (TUM), Einsteinstr. 25, 81675 Munich, Germany; 2 Department of Radiation Oncology, Klinikum rechts der isar, TUM, Munich, Germany; 3 German Cancer Consortium (DKTK), Partner Site Munich, Germany; 4 German Cancer Research Center (DKFZ), Heidelberg, Germany; 5 Department of Radiation Medicine (IRM), Helmholtz Zentrum München, Neuherberg, Germany; 6 Department of Radiooncology and Radiotherapy, Charité University Hospital Berlin, Berlin, Germany; 7 German Cancer Consortium (DKTK), Partner Site Berlin, Germany; 8 Department of Radiotherapy and Oncology, Goethe University, Frankfurt, Germany; 9 German Cancer Consortium (DKTK), Partner Site Frankfurt, Germany; 10 OncoRay – National Centre for Radiation Research in Oncology, Faculty of Medicine and University Hospital Carl Gustav Carus, Technische Universität Dresden, Helmholtz-Zentrum Dresden, Rossendorf, Dresden, Germany; 11 German Cancer Consortium (DKTK), Partner Site Dresden, Germany; 12 Department of Radiotherapy and Radiation Oncology, Faculty of Medicine, University Hospital Carl Gustav Carus, Technische Universität, Dresden, Germany; 13 National Center for Tumor Diseases (NCT), Partner Site Dresden, Germany; 14 Faculty of Medicine and University Hospital, Partner Site Dresden, Germany; 15 Carl Gustav Carus, Technische Universität Dresden, Dresden, Germany; 16 Helmholtz Association/Helmholtz-Zentrum Dresden – Rossendorf (HZDR), Dresden, Germany; 17 OncoRay, Dresden, Germany; 18 Department of Radiation Oncology, Medical Centre University of Freiburg, Freiburg, Germany; 19 German Cancer Consortium (DKTK), Partner Site Freiburg, Germany

**Keywords:** SCCHN, Prediction of locoregional recurrence, Immunophenotyping, Radiochemotherapy, Lymphocyte subpopulations, NK cell subsets

## Abstract

**Background:**

Radiochemotherapy (RCT) has been shown to induce changes in immune cell homeostasis which might affect antitumor immune responses. In the present study, we aimed to compare the composition and kinetics of major lymphocyte subsets in the periphery of patients with non-locoregional recurrent (n = 23) and locoregional recurrent (n = 9) squamous cell carcinoma of the head and neck (SCCHN) upon primary RCT.

**Methods:**

EDTA-blood of non-locoregional recurrent SCCHN patients was collected before (t0), after application of 20–30 Gy (t1), in the follow-up period 3 (t2) and 6 months (t3) after RCT. In patients with locoregional recurrence blood samples were taken at t0, t1, t2 and at the time of recurrence (t5). EDTA-blood of age-related, healthy volunteers (n = 22) served as a control (Ctrl). Major lymphocyte subpopulations were phenotyped by multiparameter flow cytometry.

**Results:**

Patients with non-recurrent SCCHN had significantly lower proportions of CD19^+^ B cells compared to healthy individuals before start of any therapy (t0) that dropped further until 3 months after RCT (t2), but reached initial levels 6 months after RCT (t3). The proportion of CD3^+^ T and CD3^+^/CD4^+^ T helper cells continuously decreased between t0 and t3, whereas that of CD8^+^ cytotoxic T cells and CD3^+^/CD56^+^ NK-like T cells (NKT) gradually increased in the same period of time in non-recurrent patients. The percentage of CD4^+^/CD25^+^/FoxP3^+^ regulatory T cells (Tregs) decreased directly after RCT, but increased above initial levels in the follow-up period 3 (t2) and 6 (t3) months after RCT. Patients with locoregional recurrence showed similar trends with respect to B, T cells and Tregs between t0 and t5. CD4^+^ T helper cells remained stably low between t0 and t5 in patients with locoregional recurrence compared to Ctrl. NKT/NK cell subsets (CD56^+^/CD69^+^, CD3^−^/CD56^+^, CD3^−^/CD94^+^, CD3^−^/NKG2D^+^, CD3^−^/NKp30^+^, CD3^−^/NKp46^+^) increased continuously up to 6 months after RCT (t0-t3) in patients without locoregional recurrence, whereas in patients with locoregional recurrence, these subsets remained stably low until time of recurrence (t5).

**Conclusion:**

Monitoring the kinetics of lymphocyte subpopulations especially activatory NK cells before and after RCT might provide a clue with respect to the development of an early locoregional recurrence in patients with SCCHN. However, studies with larger patient cohorts are needed.

***Trial registration*:**

Observational Study on Biomarkers in Head and Neck Cancer (HNprädBio), NCT02059668. Registered on 11 February 2014, https://clinicaltrials.gov/ct2/show/NCT02059668.

**Supplementary Information:**

The online version contains supplementary material available at 10.1186/s13014-021-01868-5.

## Background

Head and neck cancer is the seventh most common cancer worldwide with an incidence rate of approximately 600.000 new cases in 2012 [1]. Despite progress in radiation oncology over the past decades, the 5 year survival rate of 40–60% of patients with locally advanced SCCHN is still not satisfying [1]. In advanced inoperable stages, SCCHN patients are treated primarily with RCT. Potential prognostic markers for the response to RCT include the human papillomavirus (HPV) infection status, tumor infiltrating lymphocytes (TILs), such as CD8^+^ cytotoxic T cells and CD3^−^ NK cells, and the Hsp70 and PD-L1 expression of the tumor. These biomarkers are usually assessed in formalin-fixed, paraffin-embedded (FFPE) tumor specimens of patients who underwent radical surgery [2, 3]. Due to the lack of tumor material after RCT there is an unmet medical need for prognostic biomarkers which can be determined in the peripheral blood of patients. Radiotherapy (RT) as well as chemotherapy (CT) can result in drastic changes in immune-related parameters, such as the composition, phenotype and function of immunocompetent effector cells. Thus, the abundance and/or activation status of these markers during therapy might provide valuable tools to predict clinical outcome [4]. Therapy-induced modulations in immune cell homeostasis are of great importance as they might interfere with antitumor immune responses [5]. Therefore, we investigated the composition of major lymphocyte subsets in the peripheral blood of SCCHN patients treated with primary RCT before, during and at different time points after treatment.

## Methods

### Study collective

Thirty-two patients (n = 32), with histologically confirmed squamous cell carcinoma of the oral cavity, oro- and hypopharynx at UICC stadium III or IV without any remote metastases were included into the study (Table 1). The patients are a subset of patients recruited into the HNprädBio study (clinicaltrials.gov, NCT02059668) of the German Cancer Consortium Radiation Oncology Group (DKTK-ROG), which is based on the availability of blood samples in the time period from May 2014 till December 2016. Nine of these patients developed a locoregional recurrence within a median time period of 11 months (range: 3–15 months) after diagnosis (t0) (Table 2). All patients received primary state-of-the-art RCT with a total radiation dose of 69–72 Gy to the boost volume, > 49 Gy to the elective volume and a cisplatinum-based simultaneous CT. Directly after RCT all patients were checked radiologically for accessing tumor response. Patients who did not show tumor response after RCT are categorized as patients with “persistent tumors” and were excluded from the study. Patients with initial secondary tumors, previous RT and neoadjuvant CT were not enrolled in the HNprädBio study. Patients with missing data were excluded from the study. Patients' characteristics are represented in Tables 1 and 2.

**Table 1:** Patients’ characteristics

Total number of patients	n (%) 32 (100%)
Men	27 (84.4%)
Women	5 (15.6%)
Age (years)	59 (41–74)
*Tumor site*	
Oral cavity	11 (34.4%)
Oropharnyx	13 (40.6%)
Hypopharynx	8 (25%)
*cTNM*	
cT2	4 (12.5%)
cT3	10 (31.25%)
cT4	18 (56.25%)
cN0	2 (6.25%)
cN1	5 (15.6%)
cN2b	11 (34.4%)
cN2c	14 (43.75%)
cM0	32 (100%)
G2	17 (53.1%)
G3	12 (37.5%)
Gx	3 (9.4%)
Nicotine consumption (packyears)	30.9

**Table 2:** Characteristics of recurrent SCCHN patients

Recurrent patient no.	Age	Sex	Tumor site	TNM classification	Time of locoregional recurrence
1	56	Female	Oropharynx	cT4 cN2c cM0 G2	11
2	61	Male	Hypopharynx	cT3 cN2c cM0 G3	6
3	61	Male	Oropharynx	cT4 cN2c cM0 G2	3
4	63	Female	Oral cavity	cT4 cN2c cM0 G2	11
5	71	Male	Hypopharynx	cT3 cN2b cM0 G2	8
6	52	Male	Oropharynx	cT4 cN2c cM0 G3	9
7	67	Male	Oral cavity	cT3 cN2 cM0 G2	13
8	44	Male	Hypopharynx	cT3 cN2b cM0 G2	15
9	73	Male	Oropharynx	cT3 cN0 cM0 G2	11

*nr.* number

EDTA-blood (7.5 ml) was collected at five sequential time points: before start of RCT (t0), after application of 20–30 Gy (t1), 3 months (t2), 6 months (t3) after RCT and at time of suspicion of recurrence or metastases, and/or after histological examination (t5, 3–15 months after t0). A centralized analysis of blood samples was performed according to a standard protocol. All patients were followed-up until t3, only very few (non-recurrent and recurrent) patients had been taken an additional blood sample after 12 months (t4). Data of recurrent patients within the study period were included, whereas data of non-recurrent patients at time point t4 were not included into the study since the number was too small for statistical analysis. The follow up schedules were individually adapted to the patient`s risk factors. Generally, in the first two years after primary tumor occurrence clinical examinations were performed every 3 months and radiological diagnostics every 6 months. Then in the following three years clinical examinations were repeated every 6 months and radiological diagnostics every 12 months. In case of suspicion of tumor recurrence during the clinical examination radiological diagnostics were also performed.

As a control (Ctrl), blood samples of twenty-two age-related healthy human volunteers (n = 22) with a median age of 64 years (range: 27–80 years) were included into the study. This trial was approved by the ethics committees of all eight DKTK partner sites and all patients and healthy donors gave written informed consent.

### Flow cytometry

The following major lymphocyte subpopulations were analyzed using fluorescence-labeled antibodies (Additional file 1: Table S1) by multiparameter flow cytometry on a FACSCalibur flow cytometer (BD Biosciences):

Leukocytes (CD45).

B lymphocytes (CD3^−^/CD19^+^ B cells).

T lymphocytes (CD3^+^/CD56^−^ T cells, CD3^+^/CD4^+^ T helper cells, CD3^+^/CD8^+^ cytotoxic T cells).

T regulatory cells (CD4^+^/CD25^+^/FoxP3^+^ Tregs).

Natural killer-like T (NKT) cells (CD3^+^/CD56^+^NKT cells, activated CD56^+^/CD69^+^ NKT cells).

Natural killer (NK) cells (activated CD56^+^/CD69^+^ NK cells, CD3^−^/CD56^+^ NK cells, CD3^−^/CD94^+^ NK cells, CD3^−^/NKG2D^+^ NK cells, CD3^−^/NKp30^+^ NK cells, CD3^−^/NKp46^+^ NK cells) and NK subsets (CD56^bright/dim^/CD16 NK cells).

Briefly, 100 µl of EDTA-blood was mixed with different combinations of fluorescence-labeled, undiluted antibodies according to the manufacturer`s recommendations. After incubation in the dark for 15 min, washing in phosphate buffered saline (PBS)/10% heat-inactivated fetal calf serum (Sigma F7524) and erythrocyte lysis buffer (BD, 349,202), a total of 100.000 CD45^+^ cells were gated and analyzed. In case that CD45 was not in the antibody panel, the data were determined on the basis of at least one additional marker that is in common with a panel that contains the leukocyte marker CD45. To determine the proportion of regulatory T cells, the CD4^+^/CD25^+^ gated T cell population was stained with FoxP3 antibody after fixation (BD51-9005451) and permeabilization (BD51-9005450). The data were analyzed by using the software BD CellQuest Pro. The ratio of positively stained cells is defined as the percentage of cells within a defined lymphocyte gate minus the percentage of cells stained with a fluorescence labeled control antibody.

### Statistics

Statistical differences between sets of data were evaluated by IBM SPSS Statistics software (IBM GmbH, Ehningen, Germany). For data sets following a normal distribution the student’s t-test was used, for all other data sets the Mann–Whitney Rank Sum Test was used. Values at different time points before, during and after RCT (t0, t1, t2, t3, t5) of all patients were considered as dependent samples; values at different time points of non-recurrent and locoregional recurrent patients in comparison with healthy controls were considered as independent samples. Data sets were considered as statistically significantly different at *p* ≤ 0.05.

## Results

### ***Kinetics of the proportion of CD3***^***−***^***/CD19***^+^***B cells before, during RCT and in the follow-up period***

**Fig. 1:**
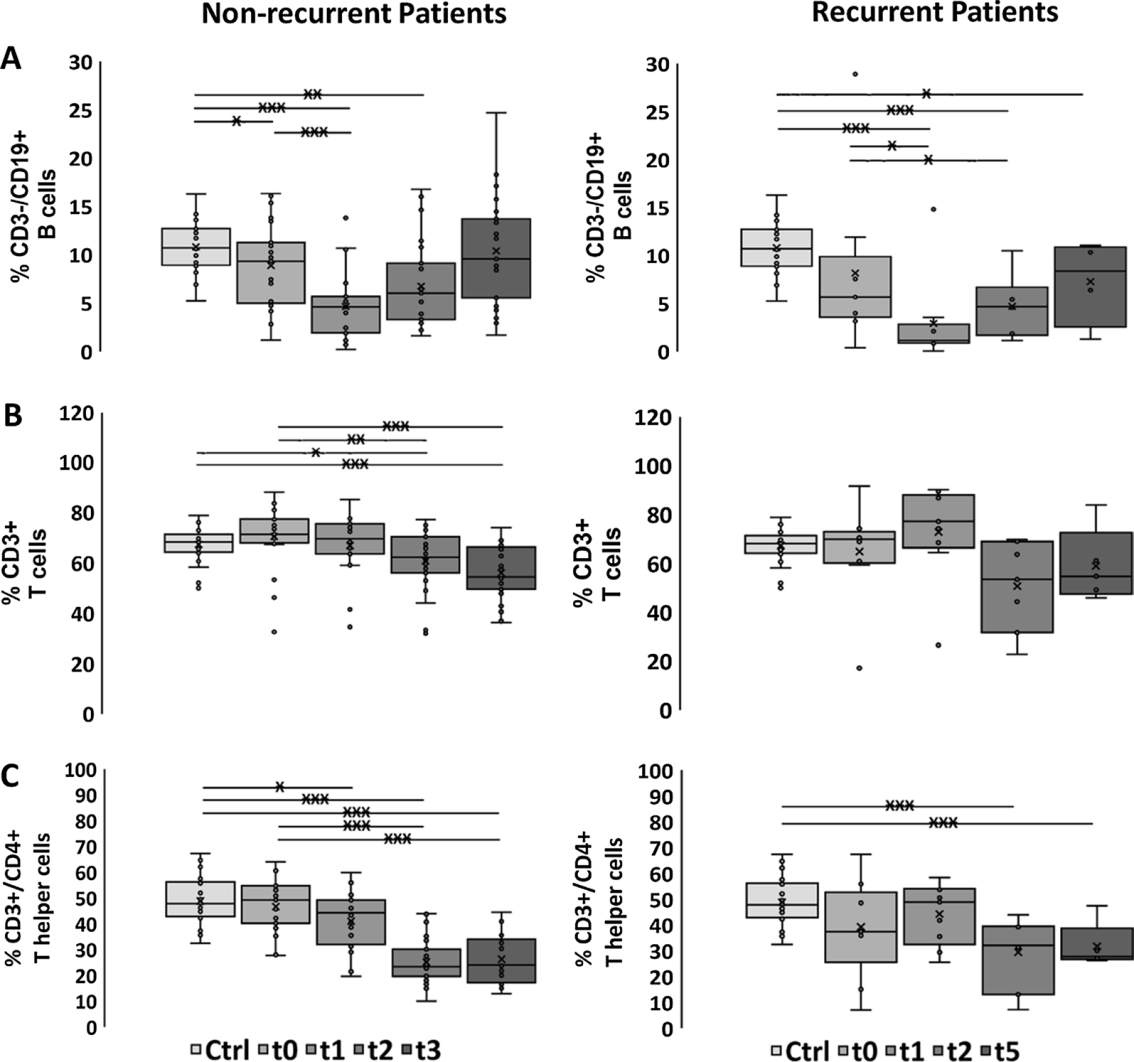

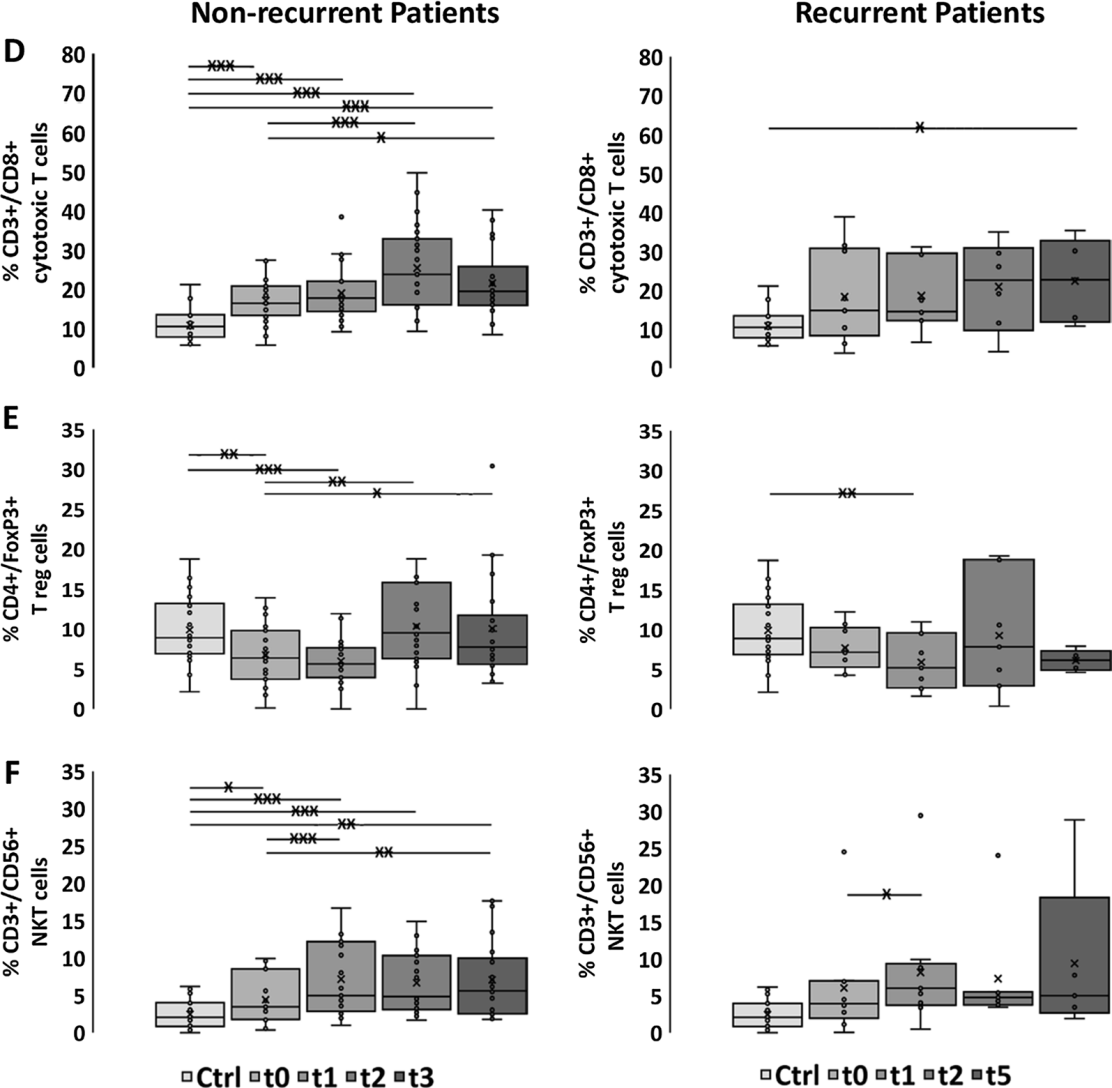
Immunophenotyping of major lymphocyte subpopulations. Percentages of **a** CD3^−^/CD19^+^ B cells, **b** CD3^+^ T cells, **c** CD3^+^/CD4^+^ T helper cells, **d** CD3^+^/CD8^+^ cytotoxic T cells, **e** CD4^+^/CD25^+^/FoxP3^+^ Tregs, **f** CD3^+^/CD56^+^ NK-like T cells in healthy controls (Ctrl, n = 22), non-recurrent (n = 23) and recurrent patients (n = 9) with SCCHN before (t0), after application of 20–30 Gy (t1), 3 months (t2), 6 months (t3) after treatment and at time of locoregional recurrence (t5, 3–15 months after t0). The data show mean values ± standard deviation of the percentage of positively stained cells. Significances are illustrated between t0 and other time points (tx) after start of RCT as well as between controls (Ctrl) and all time points of RCT (*p ≤ 0.05; **p ≤ 0.01; ***p ≤ 0.001)

As summarized in Fig. 1a and Additional file 1: Table S2, the percentage of CD3^−^/CD19^+^ B cells in patients with non-recurring SCCHN (n = 23) was significantly lower compared to that of healthy volunteers (Ctrl) already before start of RCT at t0 (t0 vs. Ctrl: 8.88% vs. 10.83%, *p* ≤ 0.05), and remained low until 3 months after RCT at t2. The most striking drop in B cells was observed directly after the application of 20–30 Gy at t1 (t0 vs. t1: 8.88% vs. 4.66%, *p* ≤ 0.001; Additional file 1: Table S3). A recovery to initial B cell levels was detected 6 months after RCT at t3 (Fig. 1a, Additional file 1: Table S2). Patients with locoregional recurrence did not reach initial levels up to t5 (3–15 months) in case of tumor recurrence (Fig. 1a, Additional file 1: Table S5).

### Kinetics of the proportions of CD3^+^/CD4^+^ T helper, CD3^+^/CD8^+^ cytotoxic and CD4^+^/CD25^+^/FoxP3^+^ regulatory T cell subsets before, during RCT and in the follow-up period

The percentage of CD3^+^/CD56^−^ T cells in SCCHN patients without locoregional recurrence dropped significantly 3 (t2) and 6 (t3) months after RCT compared to initial levels (t0 vs. t2: 70.37% vs. 60.88%, *p* ≤ 0.01; t0 vs. t3: 70.37% vs. 56.05%, *p* ≤ 0.001; Fig. 1b). At t2 and t3, the values were also significantly lower than those of controls (Ctrl vs. t2: 67.29% vs. 60.88%, *p* ≤ 0.05, Ctrl vs. t3: 67.29% vs. 56.05%, *p* ≤ 0.001; Fig. 1b, Additional file 1: Table S2) and to initial levels (Additional file 1: Table S3). In patients with locoregional recurrence T cell ratios showed a slight increase at t1 that dropped below initial levels until t5 (Fig. 1b, Additional file 1: Table S4).

As CD3^+^/CD4^+^ T helper cells make up more than 2/3 of the total CD3^+^ T cell counts, the kinetics of these cells followed that of the T cells in the course of therapy and in the follow-up period of SCCHN patients without locoregional recurrence. T helper cells dropped significantly at t2 and t3 (t0 vs. t2: 46.7% vs. 25.2%, t0 vs. t3: 46.7% vs. 26.27%, *p* ≤ 0.001; Fig. 1c, Additional file 1: Table S3), and patients without locoregional recurrence had significantly lower percentages of T helper cells compared to healthy controls between t1 and t3 (Ctrl vs. t1: 48.82% vs. 46.7%, *p* ≤ 0.05; Ctrl vs. t2: 48.82% vs. 25.2%, Ctrl vs. t3: 48.82% vs. 26.27%, *p* ≤ 0.001; Fig. 1c, Additional file 1: Table S2). Interestingly, the proportion of T helper cells in patients with locoregional recurrence was below that of controls and non-recurrent patients already before start of RCT and remained unaltered low throughout the whole course of therapy (Fig. 1c, Additional file 1: Tables S4, S5).

In contrast to the T helper cells, non-recurrent SCCHN patients had significantly higher proportions of cytotoxic CD8^+^ T cells at all time points (t0, t1, t2, t3) compared to controls (Ctrl vs. t0: 10.83% vs. 17.12%, Ctrl vs. t1: 10.83% vs. 19.03%, Ctrl vs. t2; 10.83% vs. 25.44%, Ctrl vs. t3: 10.83% vs. 10.54%, *p* ≤ 0.001; Fig. 1d, Additional file 1: Table S2). After RCT, the proportion of CD8^+^ T cells in patients without locoregional recurrence increased further (t0 vs. t2: 17.12% vs. 25.44%, *p* ≤ 0.001; t0 vs. t3: 17.12% vs. 10.54%, *p* ≤ 0.05; Fig. 1d, Additional file 1: Table S3). Notably, recurrent patients showed a trend towards higher percentages of cytotoxic T cells at all time points (Fig. 1d, Additional file 1: Tables S4 and S5), but no significant increase after RCT.

The percentage of regulatory T cells in SCCHN patients without locoregional recurrence at t0 was lower than that in healthy controls and dropped further directly after application of 20–30 Gy (t1) (Ctrl vs. t0: 9.92% vs. 6.77%, *p* ≤ 0.01; Ctrl vs. t1: 9.92% vs. 5.98%, *p* ≤ 0.001; Fig. 1e, Additional file 1: Table S2). However, in the follow-up period (t2, t3) the percentage of Tregs increased significantly above initial levels (t0 vs. t2: 6.77% vs. 10.34%, *p* ≤ 0.01; t0 vs. t3: 6.77% vs. 10.06%, *p* ≤ 0.05; Fig. 1e, Additional file 1: Table S3). In patients with locoregional recurrence Tregs remained below that of controls throughout the course of therapy (Fig. 1e, Additional file 1: Table S4).

### Kinetics of the proportion of CD3^+^/CD56^+^ NKT cells before, during RCT and in the follow-up period

Similar to CD8^+^ T cells, NK-like T (NKT) cells increased significantly during therapy in patients without locoregional recurrences (t0 vs. t1: 4.41% vs. 7.18%, *p* ≤ 0.001; t0 vs. t3: 4.41% vs. 7.07%, *p* ≤ 0.01; Fig. 1f, Additional file 1: Table S3). Notably, before start of therapy at t0, the percentage of NKT cells was almost twice as high compared to healthy controls (Ctrl vs. t0: 2.46% vs. 4.41%, *p* ≤ 0.05, Ctrl vs. t1: 2.46% vs. 7.18%, *p* ≤ 0.001; Ctrl vs. t2: 2.46% vs. 6.69%, *p* ≤ 0.001; Ctrl vs. t3: 2.46% vs. 7.07%, *p* ≤ 0.01; Fig. 1f, Additional file 1: Table S2). Patients with locoregional recurrences showed stably higher proportions of NKT cells than controls throughout the therapy (Fig. 1f, Additional file 1: Table S4).

### Kinetics of the proportion of NK cell subsets (CD56 + /CD69 + , CD3^−^/CD56^+^, CD3^−^/CD94^+^, CD3^−^/NKG2D^+^, CD3^−^/NKp30^+^, CD3^−^/NKp46^+^) and CD56^bright/dim^/CD16 NK subset analysis before, during RCT and in the follow-up period

**Fig. 2:**
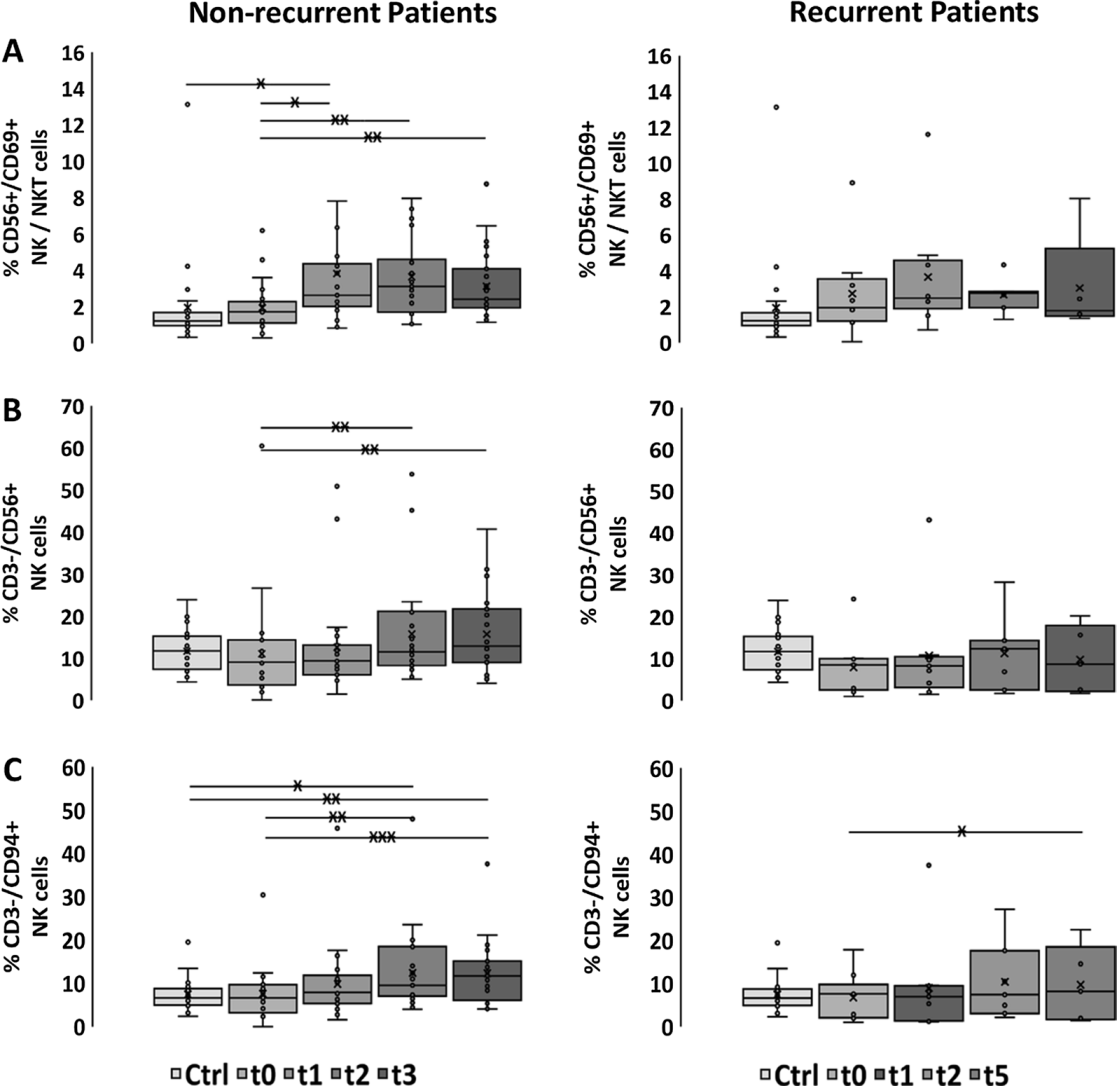

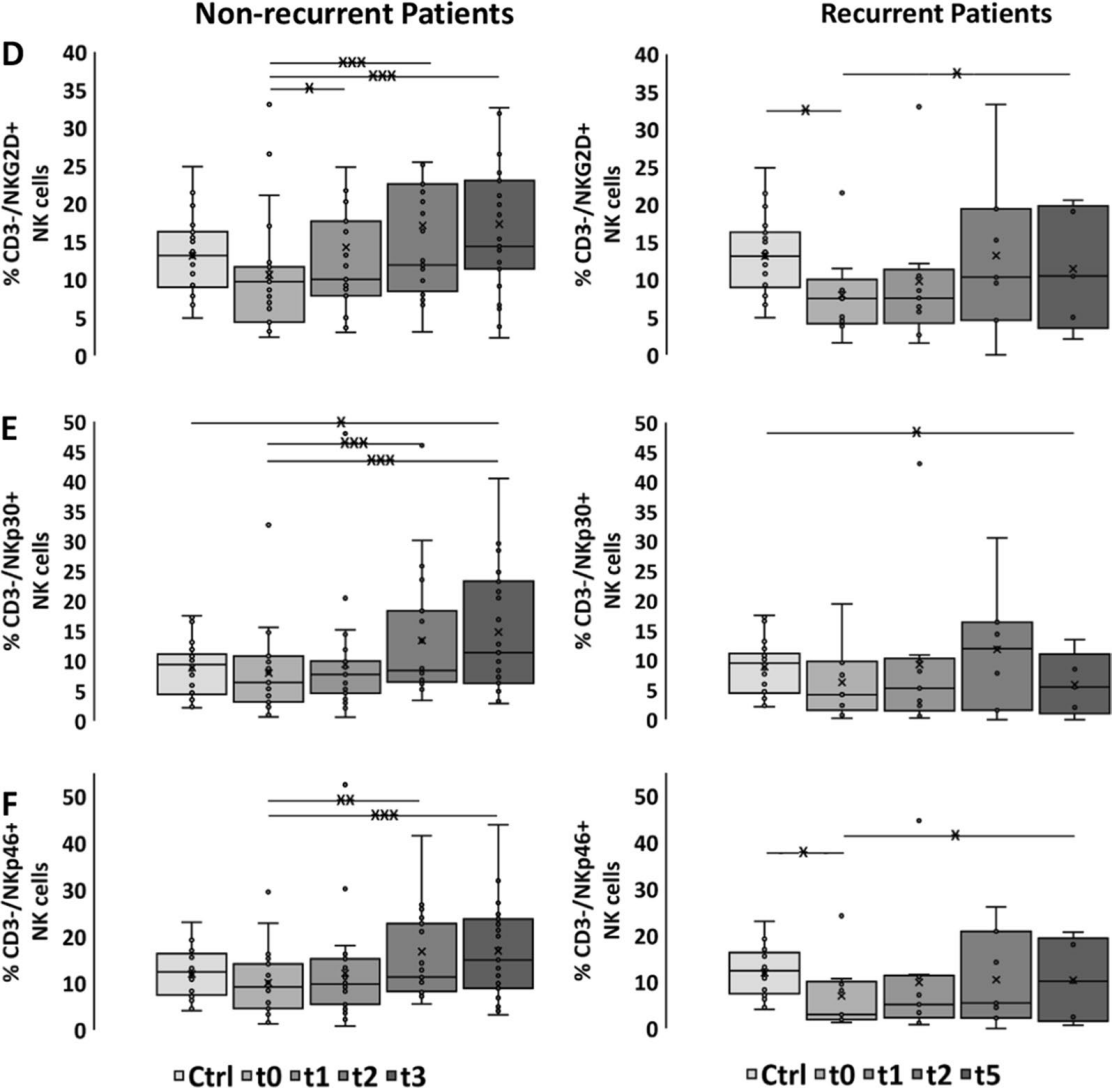
Immunophenotyping of NK cell subpopulations. Percentages of **a** CD56^+^/CD69^+^ NKT/NK cells, **b** CD3^−^/CD56^+^ NK cells, **c** CD3^−^/CD94^+^ NK cells, **d** CD3^−^/NKG2D^+^ NK cells, **e** CD3^−^/NKp30^+^ NK cells, **f** CD3^−^/NKp46^+^ NK cells in healthy controls (Ctrl, n = 22), non-recurrent (n = 23) and recurrent patients (n = 9) with SCCHN before (t0), after application of 20–30 Gy (t1), 3 months (t2), 6 months (t3) after treatment and at time of locoregional recurrence (t5, 3–15 months after t0). The data show mean values ± standard deviation of the percentage of positively stained cells. Significances are illustrated between t0 and other time points (tx) after start of RCT as well as between controls (Ctrl) and all time points of RCT (*p ≤ 0.05; **p ≤ 0.01; ***p ≤ 0.001)

Already during RCT the proportion of NK cells continuously increased up to t3 in patients without, but not in patients with locoregional recurrence (Fig. 2b). Before RCT at t0 none of the tested NK cell subpopulations differed between healthy controls and patients without locoregional recurrence (Fig. 2, Additional file 1: Table S6). However, the percentage of CD3^−^/CD56^+^ NK cells in patients without locoregional recurrence increased significantly after RCT (t0 vs. t2: 11.1% vs. 15.83%, t0 vs. t3: 11.1% vs. 15.79%, *p* ≤ 0.01; Fig. 2b, Additional file 1: Table S7) In contrast, patients with recurrence had lower proportions of all NK cell subpopulations already before RCT than controls that remained at low levels at all other time points (Fig. 2b–f, Additional file 1: Tables S8, S9).

Overall, the percentages of all NK cell subsets increased significantly after RCT in patients without locoregional recurrences (Fig. 2a–f, Additional file 1: Table S7). At t3, values of all NK cell subsets including those bearing activating receptors such as NKG2D, NKp30 and NKp46 were significantly higher than initial levels and those of healthy controls (Fig. 2a–f, Additional file 1: Tables S6, S7). In contrast, all NK cell subgroups in recurrent patients remained below that of patients without locoregional recurrence (Fig. 2a–f, Additional file 1: Table S8). Furthermore, recurrent patients had lower NK cell counts at time of locoregional recurrence (t5) compared to patients without recurrence at t3 (Fig. 2a–f, Additional file 1: Table S8).

A subgroup analysis of CD56^bright^/CD16^+^ NK cells in SCCHN patients without locoregional recurrence revealed significantly higher proportions of CD56^bright^/CD16^+^ NK cells compared to healthy controls, which dropped after application of 20–30 Gy at t1, but increased until t3 (Table 3). A similar course was observed with respect to the CD56^bright^/CD16-NK cell subset in patients without locoregional recurrence. In recurrent patients both CD56^bright^ NK cell subsets remained below that of patients without recurrence throughout the whole course of therapy (t0–t5) (Table 4). In contrast, values of the CD56^dim^ NK cell subset appeared to be elevated in recurrent patients (Table 4).

**Table 3:** Significantly different values in NK cell subsets in controls (Ctrl) and non-recurrent SCCHN patients

%	Ctrl	t0	t1	t2	t3
CD56^bright^/CD16- NK cells	1.83 ± 1.14	2.48 ± 1.94	1.78 ± 1.65*	2.11 ± 1.55	2.25 ± 1.62
CD56^bright^/CD16^+^ NK cells	2.52 ± 1.34	4.72 ± 3.98*	3.72 ± 3.32	3.08 ± 2.15	4.09 ± 2.68
CD56^dim^/CD16^−^ NK cells	2.8 ± 1.8	3.98 ± 2.82	4.42 ± 3.53	2.32 ± 1.33	7.5 ± 8.53
CD56^dim^/CD16^+^ NK cells	87.27 ± 5.97	83.10 ± 10.29	84.93 ± 10.49	89.3 ± 5.46*	82.8 ± 12.27
CD56^−^/CD16^+^ NK cells	0 ± 0	0.01 ± 0.02	0.14 ± 0.27	0.05 ± 0.09	0.09 ± 0.16

The significance is between the two underlined values. E.g. significance in CD56bright/CD16- NK cells between t0 and t1 is *p<=0.05

Composition of NK cell subsets (CD56/CD16) in % (mean value ± standard deviation) in healthy controls (Ctrl, n = 22) and non-recurrent patients (n = 23) before (t0), after application of 20–30 Gy (t1), 3 months (t2) and 6 months (t3) after RCT. Significantly different values (t0 vs. t1; Ctrl vs. t0; t0 vs. t2) are indicated in bold with an asterisk (*p ≤ 0.05; **p ≤ 0.01; ***p ≤ 0.001)

**Table 4:** Significantly different values in NK cell subsets in controls (Ctrl) and recurrent SCCHN patients

%	Ctrl	t0	t1	t2	t5
CD56^bright^/CD16^−^ NK cells	1.83 ± 1.14	0.92 ± 0.7*	0.75 ± 0.63*	2.07 ± 1.52	1.6 ± 1.52
CD56^bright^/CD16^+^ NK cells	2.52 ± 1.34	2.88 ± 1.85	1.47 ± 0.7*	5.76 ± 4.85	1.54 ± 1.32
CD56^dim^/CD16^−^ NK cells	2.8 ± 1.8	10.19 ± 11.85	6.08 ± 5.3	4.3 ± 3.97	2.25 ± 2.05
CD56^dim^/CD16^+^ NK cells	87.27 ± 5.97	81.4 ± 13.9	88.23 ± 7.52	84.78 ± 11.56	92.88 ± 4.26*
CD56^−^/CD16^+^ NK cells	0 ± 0	0 ± 0	0 ± 0	0 ± 0	0 ± 0

The significance is between the two underlined values. E.g. significance in CD56bright/CD16- NK cells between t0 and t1 is *p<=0.05

Composition of NK cell subsets (CD56/CD16) in % (mean value ± standard deviation) in healthy controls (Ctrl, n = 22) and recurrent patients (n = 9) before (t0), after application of 20–30 Gy (t1), 3 months (t2) after RCT and at time of locoregional recurrence (t5, 3–15 months after t0). Significantly different values (Ctrl vs. t0, t1; t0 vs. t1; Ctrl vs. t5) are indicated in bold with an asterisk (*p ≤ 0.05; **p ≤ 0.01; ***p ≤ 0.001)

## Discussion

It has been reported that the amount of B cells that contribute to the humoral immune response are lower in SCCHN patients than in healthy individuals [6–8]. Belka et al. were among the first to describe B cells as the most sensitive lymphocyte subpopulation towards radiation since they rapidly undergo radiation-induced apoptosis. However, the drop in B cells was not associated with a decline in the immunoglobulin levels [9], which might provide a hint that B cell activity is not impaired by radiotherapy. Our data are in line with these previous findings showing a significant drop in B cells after application of 20–30 Gy during RCT and a recovery to initial levels within a time period of 6 months. The recovery might be mediated by B cell precursor cells originating from non-irradiated bone marrow [10] or by a retranslocation of B cells into the periphery. However, further studies are needed to address the question whether B cell functions such as antibody production or antigen presenting capacity in secondary lymphoid organs are impaired in SCCHN patients following RCT.

A long-lasting T cell imbalance with diminished T cell counts can be caused by immunosuppressive effects of the tumor and its microenvironment or by treatment-related effects [11]. Abnormalities in the T cell repertoire have been described for patients with myeloma, breast-, ovarian- and liver cancer [12–15]. Similar to our data, Nollert et al. demonstrated a significant drop in total CD3^+^ T cell counts in stage IV SCCHN patients by RT combined with a platinum-based chemotherapy [16]. Since cisplatinum is known to cause a systemic toxicity which is associated with a reduced T cell proliferation, the drug (prescribed cumulative total dose of cisplatinum was not less than 180 mg/m^2^ body surface area) might be responsible for the steady decrease in CD3^+^ T cells during and after RCT in our cohort [17, 18].

Other studies have reported that the CD4^+^ T cell counts in SCCHN patients, especially in advanced UICC stages III and IV, are significantly lower than in healthy subjects [19, 20]. Melioli et al. observed a severe proliferative defect especially in the CD4^+^ T helper subset [21]. As T helper cells are the dominant T cell subset (up to 2/3 of the total T cell counts) and cisplatin’s systemic toxicity results in a reduced T cell proliferation [17, 18], the drop in CD3^+^ T cells is reflected in the CD4^+^ T helper subpopulation upon RCT. In line with our findings, others also observed a decrease in the T helper subset in head and neck cancer patients during and after definite and adjuvant RT [11, 15, 22]. As patients with recurrent SCCHN are more likely to display abnormalities in their T cell development [23], the drop in CD4^+^ T cells before start of therapy (t0) appeared to be more pronounced in patients with locoregional recurrence compared to patients who remained free of recurrence [22–24]. Therefore, we hypothesize that low initial T helper counts might provide a potential predictive marker for patients that more likely will develop a recurrent disease.

A significant increase in the percentage of CD8^+^ T cells in SCCHN patients without locoregional recurrence upon RCT might be indicative for the induction of an antitumor immune response induced by RCT. Wolf et al. also demonstrated a weak increase in CD8^+^ T cells in head and neck cancer patients during and after definite and adjuvant RT [15]. Moreover, Balermpas et al. reported that elevated numbers of CD8^+^ tumor infiltrating lymphocytes (TILs) in SCCHN patients treated with adjuvant RCT serve as a prognostic marker for improved clinical outcome [2]. Compared to controls, SCCHN patients showed significantly increased activation status of CD4^+^ and CD8^+^ T cells, as well as a higher migratory potential of these lymphocyte populations [24], which might explain an increased infiltration into the tumor tissue. However, an expansion of CD8^+^ effector cells was accompanied by a rapid demise through apoptosis [25, 26]. In line with our findings, Johnson et al. observed that patients with recurrent SCCHN tended to have higher proportions of circulating CD8^+^ cytotoxic T cells at diagnosis than patients who remained disease-free [23]. In addition, increased CD8^+^ T cells in patients at diagnosis directly correlated with the level of tumor cell differentiation and histological grading [8]. However, we could demonstrate that the proportion of CD8^+^ T cells did not increase significantly upon therapy in patients with locoregional recurrence.

While a high infiltration of FoxP3^+^ Tregs at the tumor site correlates with an impaired overall survival in patients with melanomas, cervical-, renal- and breast cancers an opposite effect was observed in colorectal, head and neck as well as esophageal cancers [27]. As head and neck cancer, colon cancer and hematologic malignancies are among those tumors which are heavily infiltrated by immune cells that facilitate tumor progression by producing growth factors and/or proinflammatory cytokines, Tregs might limit tumor-promoting inflammation by the release of immunosuppressive cytokines [28–31]. Similar to our results, Lee et al. observed a weak decline in CD4^+^/CD25^+^/FoxP3^+^ Tregs in patients with oral squamous cell carcinoma compared to healthy controls [24], while a rise in the frequency of Tregs was found in patients with SCCHN treated with adjuvant RCT [5, 32]. Finally, Kachikwu et al. observed an increase in Tregs after radiation which was associated with a more radioresistant phenotype [33].

Kobayashi et al. described the ability of radiotherapy to improve proliferation and antitumor immunity of NKT cells [34]. In our SCCHN cohort, especially patients with locoregional recurrences, exhibited increased NKT cell levels at diagnosis which further increase throughout therapy as compared to controls. These data suggest the presence of a predefined immune imbalance which correlates with treatment response and the ability of RCT to boost SCCHN patients’ immunocompetence. Additionally, the particularly high NKT cell value in recurrent patients at t0 could provide a hint for an increased risk for an early recurrence.

In our study, a significant increase in the percentage of NK cells with activating receptors such as NKG2D, NKp30 and NKp46 might reflect the effect of RCT on patients’ immunocompetence to improve antitumor responsiveness. Moreover, it also has been reported that cytotoxic lymphocytes, including CD8^+^ T cells and NK cells, contribute to the efficacy of certain chemotherapeutic drugs [4], such as cisplatinum, paclitaxel and doxorubicin, which have been shown to enhance the NK and CD8^+^ T cell-mediated killing as they increase the permeability of tumor cells towards the apoptosis inducing serine protease granzyme B [35]. Recurrent patients, in particular at diagnosis, tended to have much lower NK cell counts than healthy controls and patients without recurrence at all time points. An impaired first line defense might indicate an imbalance of the innate immune cell function in patients with recurrence.

CD56^bright^/CD16^+^ NK cells are considered to have a low cytotoxic activity and a high cytokine/chemokine production capacity, whereas the CD56^dim^/CD16^+^ NK cell population is associated with a high cytotoxic potential [36–38]. In accordance with this hypothesis, Mamessier et al. observed an increased proportion of CD56^bright^/CD16^+^ circulating NK cells in advanced breast cancer patients and patients with metastases [39]. However, more recent data by the group of Wagner et al. revealed that upon stimulation CD56^bright^/CD16^+^ NK cells can also exert direct antitumor activity [40]. CD56^bright^/CD16^+^ NK cells mediate strong cytotoxicity against MHC class I negative as well as MHC class I positive tumors [38]. Our findings indicate significant higher percentages of CD56^bright^/CD16^+^ NK cells in patients without locoregional recurrence compared to healthy controls and patients with recurrence. These values remained elevated in patients without locoregional recurrence throughout the course of therapy and in the follow-up period. Activation of NK/NKT cells is associated with an increase in the early activation marker CD69 in patients without locoregional recurrence. A high NK cell activity has been shown to correlate with lower incidence of tumors and thereby improves, alongside with NK cell infiltration in SCCHN, patients’ clinical outcome [41, 42]. Similar to our findings, Takeuchhi et al. demonstrated that a low NK cell activity leads to a higher incidence of tumor occurrence and metastasis, and its degree correlates with an increased invasiveness of the tumor [43]. In line with these findings, impairment of NK cell function is greater in patients with advanced tumors, as primary squamous cell carcinoma patients in stages T3-T4 showed lower NK cell activity than patients in earlier stages T1-T2 [44, 45]. Ye et al. observed a significant decrease in CD56^+^/CD16^+^ circulating NK cells in oral cancer patients after surgery [46], indicating that not only RCT, but also inflammatory effects induced by surgery may have an impact on the NK cell hemostasis.

In our study, the proportion of all activated NK cell subsets was lower in the recurrent patient group compared to healthy individuals and patients without locoregional recurrence. Activatory NK cell subsets increased significantly in the course of therapy in non-recurrent patients, but remained nearly unaltered until t5 in patients with locoregional recurrence. Since NK cell responses occur fast this finding might be of relevance for predicting a potential recurrence and to adjust the treatment at an earlier time point. Our findings demonstrated the ability of RCT in enhancing antitumor immune responses mediated by NK cells in patients with SCCHN. Therefore, we speculate that a combination of RCT with immunotherapy such as immune checkpoint inhibitors might provide a promising strategy to increase antitumor activity of NK cells in patients with SCCHN after RCT who are on high risk to develop recurrences.

## Conclusions

We could show altered lymphocyte compositions in patients with non-locoregional recurrences and patients with locoregional recurrences after primary RCT. These changes already existed before start of therapy and persisted for months in the follow-up period. B cells, T cells, T helper cells and regulatory T cells dropped significantly after RCT, but unlike T and T helper cells, B cells and regulatory T cells recovered to initial levels 6 months after RCT. Inversely, cytotoxic T cells, NKT and especially activating CD3^−^ NK cell subsets gradually increased in the same time period in non-recurrent patients, but remained nearly unaltered in patients with recurrent tumors. Following validation of our data in a larger patient cohort, monitoring cellular immunity in the peripheral blood of SCCHN patients during therapy might offer an opportunity for an early evaluation of treatment outcome and might help to stratify for patients that most likely will benefit from an additive immunotherapy.

## Supplementary Information

**Additional file 1**. Supplementary materials.

## Data Availability

Data and material are available by the corresponding author.

## Abbreviations

SCCHN: Squamous cell carcinoma of the head and neck
RCT: Radiochemotherapy
CT: Chemotherapy
RT: Radiotherapy
NK: Natural killer cells
NKT: Natural killer-like T cells
MHC: Major histocompatibility complex

## References

[1] Ferlay J, Soerjomataram I, Dikshit R, Eser S, Mathers C, Rebelo M (2015). Cancer incidence and mortality worldwide: sources, methods and major patterns in GLOBOCAN 2012. Int J Cancer.

[2] Balermpas P, Rödel F, Rödel C, Krause M, Linge A, Lohaus F (2016). CD8+ tumour-infiltrating lymphocytes in relation to HPV status and clinical outcome in patients with head and neck cancer after postoperative chemoradiotherapy: A multicentre study of the German cancer consortium radiation oncology group (DKTK-ROG): CD8+ TILs in SCHNN. Int J Cancer.

[3] Stangl S, Tontcheva N, Sievert W, Shevtsov M, Niu M, Schmid TE (2018). Heat shock protein 70 and tumor-infiltrating NK cells as prognostic indicators for patients with squamous cell carcinoma of the head and neck after radiochemotherapy: a multicentre retrospective study of the German Cancer Consortium Radiation Oncology Group (DKTK-ROG). Int J Cancer.

[4] Coffelt SB, de Visser KE (2015). Immune-mediated mechanisms influencing the efficacy of anticancer therapies. Trends Immunol.

[5] Schuler PJ, Harasymczuk M, Schilling B, Saze Z, Strauss L, Lang S, et al. Effects of adjuvant chemoradiotherapy on the frequency and function of regulatory T cells in patients with head and neck cancer. Clin Cancer Res. 2013;19. 10.1158/1078-0432.CCR-13-0900 PMC3855337 24097865

[6] Kaffenberger W, Hölzer-Müller L, Auberger T, Clasen BP, Hohlmeier G, van Beuningen D (1995). An immunological outcome predictive score for head and neck carcinoma patients. Strahlenther Onkol.

[7] Andrade MC, Ferreira SBP, Gonçalves LC, De-Paula AMB, de Faria ES, Teixeira-Carvalho A (2013). Cell surface markers for T and B lymphocytes activation and adhesion as putative prognostic biomarkers for head and neck squamous cell carcinoma. Hum Immunol.

[8] Boucek J, Mrkvan T, Chovanec M, Kuchar M, Betka J, Boucek V (2010). Regulatory T cells and their prognostic value for patients with squamous cell carcinoma of the head and neck. J Cell Mol Med.

[9] Belka C, Ottinger H, Kreuzfelder E, Weinmann M, Lindemann M, Lepple-Wienhues A (1999). Impact of localized radiotherapy on blood immune cells counts and function in humans. Radiother Oncol.

[10] Sage EK, Schmid TE, Sedelmayr M, Gehrmann M, Geinitz H, Duma MN (2016). Comparative analysis of the effects of radiotherapy versus radiotherapy after adjuvant chemotherapy on the composition of lymphocyte subpopulations in breast cancer patients. Radiother Oncol.

[11] Kuss I, Hathaway B, Ferris RL, Gooding W, Whiteside TL (2004). Decreased absolute counts of T lymphocyte subsets and their relation to disease in squamous cell carcinoma of the head and neck. Clin Cancer Res.

[12] Kay NE, Leong TL, Bone N, Vesole DH, Greipp PR, Ness BV (2001). Blood levels of immune cells predict survival in myeloma patients: results of an Eastern Cooperative Oncology Group phase 3 trial for newly diagnosed multiple myeloma patients. Blood.

[13] Melichar B, Tousková M, Solichová D, Králicková P, Kopecký G (2001). CD4+ T-lymphocytopenia and systemic immune activation in patients with primary and secondary liver tumours. Scand J Clin Lab Invest.

[14] Schröder W, Vering A, Stegmüller M, Strohmeier R (1997). Lymphocyte subsets in patients with ovarian and breast cancer. Eur J Gynaecol Oncol.

[15] Wolf GT, Amendola BE, Diaz R, Lovett EJ, Hammerschmidt RM, Peterson KA. Definite vs adjuvant radiotherapy. Comparative effects on lymphocyte subpopulations in patients with head and neck squamous carcinoma. Arch Otolaryngol. 1985;111:716–26. 10.1001/archotol.1985.00800130048004 2932094

[16] Nollert J, Rudat V, Daniel V, Maier H, Dietz A (1999). Einfluß der primären Radiochemotherapie auf zelluläre und subzelluläre immunologische Parameter. HNO.

[17] Garzetti GG, Ciavattini A, Provinciali M, Valensise H, Romanini C, Fabris N (1994). Influence of neoadjuvant polychemotherapy on natural killer cell activity in patients with locally advanced cervical squamous carcinoma. Gynecol Oncol.

[18] Sfikakis PP, Souliotis VL, Katsilambros N, Markakis K, Vaiopoulos G, Tsokos GC (1996). Downregulation of interleukin-2 and apha-chain interleukin-2 receptor biosynthesis by cisplatin in human peripheral lymphocytes. Clin Immunol Immunopathol.

[19] Chikamatsu K, Sakakura K, Whiteside TL, Furuya N (2007). Relationships between regulatory T cells and CD8+ effector populations in patients with squamous cell carcinoma of the head and neck. Head Neck.

[20] Bose A, Chakraborty T, Chakraborty K, Pal S, Baral R (2008). Dysregulation in immune functions is reflected in tumor cell cytotoxicity by peripheral blood mononuclear cells from head and neck squamous cell carcinoma patients. Cancer Immun.

[21] Melioli G, Semino C, Margarino G, Mereu P, Scala M, Cangemi G (2003). Expansion of natural killer cells in patients with head and neck cancer: detection of “noninhibitory” (activating) killer Ig-like receptors on circulating natural killer cells. Head Neck.

[22] Kuss I, Hathaway B, Ferris RL, Gooding W, Whiteside TL (2005). Imbalance in absolute counts of T lymphocyte subsets in patients with head and neck cancer and its relation to disease. Adv Otorhinolaryngol.

[23] Johnson JT, Rabin BS, Hirsch B, Thearle PB (1984). T-cell subpopulations in head and neck carcinoma. Otolaryngol Head Neck Surg.

[24] Lee JJ, Lin CL, Chen THH, Kok SH, Chang MC, Jeng JH (2010). Changes in peripheral blood lymphocyte phenotypes distribution in patients with oral cancer/oral leukoplakia in Taiwan. Int J Oral Maxillofac Surg.

[25] Kuss I, Donnenberg AD, Gooding W, Whiteside TL (2003). Effector CD8+CD45RO−CD27−T cells have signalling defects in patients with squamous cell carcinoma of the head and neck. Br J Cancer.

[26] Tsukishiro T, Donnenberg AD, Whiteside TL (2003). Rapid turnover of the CD8(+)CD28(-) T-cell subset of effector cells in the circulation of patients with head and neck cancer. Cancer Immunol Immunother.

[27] Shang B, Liu Y, Jiang S, Liu Y (2015). Prognostic value of tumor-infiltrating FoxP3+ regulatory T cells in cancers: a systematic review and meta-analysis. Sci Rep.

[28] Badoual C, Hans S, Rodriguez J, Peyrard S, Klein C, Agueznay NEH (2006). Prognostic value of tumor-infiltrating CD4+ T-cell subpopulations in head and neck cancers. Clin Cancer Res.

[29] Schottelius AJ, Dinter H (2006). Cytokines, NF-kappaB, microenvironment, intestinal inflammation and cancer. Cancer Treat Res.

[30] Carreras J, Lopez-Guillermo A, Fox BC, Colomo L, Martinez A, Roncador G (2006). High numbers of tumor-infiltrating FOXP3-positive regulatory T cells are associated with improved overall survival in follicular lymphoma. Blood.

[31] Jie H-B, Gildener-Leapman N, Li J, Srivastava RM, Gibson SP, Whiteside TL (2013). Intratumoral regulatory T cells upregulate immunosuppressive molecules in head and neck cancer patients. Br J Cancer.

[32] Strauss L, Bergmann C, Gooding W, Johnson JT, Whiteside TL (2007). The frequency and suppressor function of CD4+CD25highFoxp3+ T cells in the circulation of patients with squamous cell carcinoma of the head and neck. Clin Cancer Res.

[33] Kachikwu EL, Iwamoto KS, Liao Y-P, DeMarco JJ, Agazaryan N, Economou JS (2011). Radiation enhances regulatory T cell representation. Int J Radiat Oncol Biol Phys.

[34] Kobayashi K, Tanaka Y, Horiguchi S, Yamamoto S, Toshinori N, Sugimoto A (2010). The effect of radiotherapy on NKT cells in patients with advanced head and neck cancer. Cancer Immunol Immunother.

[35] Ramakrishnan R, Assudani D, Nagaraj S, Hunter T, Cho H-I, Antonia S (2010). Chemotherapy enhances tumor cell susceptibility to CTL-mediated killing during cancer immunotherapy in mice. J Clin Investig.

[36] Cooper MA, Fehniger TA, Caligiuri MA (2001). The biology of human natural killer-cell subsets. Trends Immunol.

[37] Colonna M, Navarro F, Bellón T, Llano M, García P, Samaridis J (1997). A common inhibitory receptor for major histocompatibility complex class I molecules on human lymphoid and myelomonocytic cells. J Exp Med.

[38] Takahashi E, Kuranaga N, Satoh K, Habu Y, Shinomiya N, Asano T (2007). Induction of CD16+ CD56bright NK cells with antitumour cytotoxicity not only from CD16- CD56bright NK Cells but also from CD16- CD56dim NK cells. Scand J Immunol.

[39] Mamessier E, Pradel LC, Thibult M-L, Drevet C, Zouine A, Jacquemier J (2013). Peripheral blood NK cells from breast cancer patients are tumor-induced composite subsets. J Immunol.

[40] Wagner JA, Rosario M, Romee R, Berrien-Elliott MM, Schneider SE, Leong JW, et al. CD56bright NK cells exhibit potent antitumor responses following IL-15 priming. J Clin Investig. 127:4042–58. 10.1172/JCI90387 PMC5663359 28972539

[41] Schleypen JS, Baur N, Kammerer R, Nelson PJ, Rohrmann K, Gröne EF (2006). Cytotoxic markers and frequency predict functional capacity of natural killer cells infiltrating renal cell carcinoma. Clin Cancer Res.

[42] Vivier E, Ugolini S, Blaise D, Chabannon C, Brossay L (2012). Targeting natural killer cells and natural killer T cells in cancer. Nat Rev Immunol.

[43] Takeuchi H, Maehara Y, Tokunaga E, Koga T, Kakeji Y, Sugimachi K (2001). Prognostic significance of natural killer cell activity in patients with gastric carcinoma: a multivariate analysis. Am J Gastroenterol.

[44] Vinzenz K, Micksche M (1986). Systemic and regional natural cytotoxicity in patients with head and neck cancer. J Maxillofac Surg.

[45] Wang JM (1989). Study of NK cell activity in patients with oral maxillofacial squamous cell carcinoma. Zhonghua Kou Qiang Yi Xue Za Zhi.

[46] Ye D, Xu Q, Chen W, Zhu H, Lin G, Jiang C, et al. Immunodetections and analysis of peripheral blood for patients with oral cancer undergoing operation. Shanghai Kou Qiang Yi Xue. 2004;13:83–6. 15133544

