# Supplementary material for: Comparison of the composition of lymphocyte subpopulations in non-relapse and relapse patients with squamous cell carcinoma of the head and neck before, during radiochemotherapy and in the follow-up period: a multicenter prospective study of the German Cancer Consortium Radiation Oncology Group (DKTK-ROG)
Source: Radiat Oncol. 2021 Jul 31;16:141. doi: 10.1186/s13014-021-01868-5 (PMC8325802; doi:10.1186/s13014-021-01868-5)
Supplement: Supplementary file 1 — Additional file 1. Supplementary materials. [file 13014_2021_1868_MOESM1_ESM.docx]

**Supplementary Materials**

**Supplementary Table 1:** Antibody panel, company and used concentrations

| **Specificity** | **Antibody** | **Company** | **Cat. No.** | **Concentration**  **(µl/per 100.000 cells)** |
| --- | --- | --- | --- | --- |
| **Isotype** | IgG1-FITC | BD | 345815 | 5 |
|  | IgG1-PE | BD | 345816 | 5 |
|  | IgG1-PerCP | BD | 345817 | 5 |
|  | IgG1-APC | Invitrogen | MG 105 | 1 |
| **Leukocyte** | CD45-APC | Invitrogen | MHCD4505 | 1 |
| **B** | CD19-PE | BD | 555413 | 20 |
| **T** | CD3-PerCP | BD | 345766 | 10 |
| **T cytotoxic** | CD8-PE | BD | 555366 | 20 |
| **T helper** | CD4-FITC | BD | 555346 | 20 |
| **NK/NKT** | CD56-PE | BD | 345811 | 5 |
| **NK/NKT** | CD56-FITC | BD | 345811 | 5 |
| **NK/NKT** | CD94-FITC | BD | 555888 | 5 |
| **NK** | NKG2D-PE | R&D | FAB139P | 10 |
| **NK** | NKp30-PE | BC | PN IM 3709 | 10 |
| **NK** | NKp46-PE | BC | PN IM 3711 | 10 |
| **Activation** | CD69-APC | BD | 340560 | 5 |
| **Treg** | CD4-FITC | BD | 555346 | 20 |
| **Treg** | CD3-PerCP | BD | 345766 | 10 |
| **Treg** | CD25-APC | BD | 340907 | 5 |
| **Treg** | FoxP3-PE | BD | 340907 | 5 |

Abbreviations: B, B cells; NK, NK cells; NKT, NK-like T cells; T, T cells; Tregs, regulatory T cells; BD, Becton Dickinson Biosciences; BC, Beckmann Coulter

Supplementary Table 2: Significantly different values in lymphocyte subsets in controls (Ctrl) vs. non-recurrent SCCHN patients

| % | Ctrl | t0 | t1 | t2 | t3 |
| --- | --- | --- | --- | --- | --- |
| B cells  CD3^-^/CD19^+^ | 10.83 ± 2.71 | **8.88 ± 4.29*** | **4.66 ± 3.38***** | **6.75 ± 4.47**** | 10.41 ± 5.63 |
| T cells  CD3^+^/CD56^-^ | 67.29 ± 7.41 | 70.37 ± 8 | 66.8 ± 9.31 | **60.88 ± 9.51*** | **56.05 ± 9.04***** |
| T helper cells  CD3^+^/CD4^+^ | 48.82 ± 9.24 | 46.7 ± 8.36 | **41.04 ± 9.02*** | **25.2 ±7.14***** | **26.27 ± 8.16***** |
| Cytotoxic T cells  CD3^+^/CD8^+^ | 10.83 ± 3.81 | **17.12 ± 4.46***** | **19.03 ± 5.22***** | **25.44 ± 9.22***** | **10.54 ± 6.85***** |
| Tregs  CD3^+^/CD4^+^  CD25^+^/FoxP3^+^ | 9.92 ± 4.19 | **6.77 ± 3.08**** | **5.98 ± 2.14***** | 10.34 ± 4.03 | 10.06 ± 4.73 |
| NKT cells  CD3^+^/CD56^+^ | 2.46 ± 1.95 | **4.41 ± 2.84*** | **7.18 ± 4.25***** | **6.69 ± 3.64***** | **7.07 ± 4.23**** |

Composition of lymphocyte subpopulations in % (mean value ± standard deviation) in healthy controls (Ctrl, n=22) and non-recurrent SCCHN patients (n=23) before (t0), after application of 20 to 30 Gy (t1), 3 months (t2) and 6 months (t3) after RCT. Significantly different values (ctrl vs. tx) are indicated in bold with an asterisk (*p ≤ 0.05; **p ≤ 0.01; ***p ≤ 0.001).

Supplementary Table 3: Significantly different values in lymphocyte subsets in the course of treatment (t0 vs. tx) in non-recurrent SCCHN patients

| % | t0 | t1 | t2 | t3 |
| --- | --- | --- | --- | --- |
| B cells  CD3^-^/CD19^+^ | 8.88 ± 4.29 | **4.66 ± 3.38***** | 6.75 ± 4.47 | 10.41 ± 5.63 |
| T cells  CD3^+^/CD56^-^ | 70.37 ± 8 | 66.8 ± 9.31 | **60.88 ± 9.51**** | **56.05 ± 9.04***** |
| T helper cells  CD3^+^/CD4^+^ | 46.7 ± 8.36 | **41.04 ± 9.02*** | **25.2 ±7.14***** | **26.27 ± 8.16***** |
| Cytotoxic T cells  CD3^+^/CD8^+^ | 16.46 ± 5.05 | 17.12 ± 4.46 | **19.03 ± 5.22***** | **25.44 ± 9.22*** |
| Tregs  CD3^+^/CD4^+^  CD25^+^/FoxP3^+^ | 6.77 ± 3.08 | 5.98 ± 2.14 | **10.34 ± 4.03**** | **10.06 ± 4.73*** |
| NKT cells  CD3^+^/CD56^+^ | 4.2 ± 2.61 | **4.41 ± 2.84***** | **7.18 ± 4.25**** | **6.69 ± 3.64**** |

Composition of lymphocyte subpopulations in % (mean value ± standard deviation) in non-recurrent SCCHN patients (n=23) before (t0), after application of 20 to 30 Gy (t1), 3 months (t2) and 6 months (t3) after RCT. Significantly different values (t0 vs. tx) are indicated in bold with an asterisk (*p ≤ 0.05; **p ≤ 0.01; ***p ≤ 0.001).

Supplementary Table 4: Significantly different values in lymphocyte subsets in controls (Ctrl) vs. recurrent SCCHN patients

| % | Ctrl | t0 | t1 | t2 | t5 |
| --- | --- | --- | --- | --- | --- |
| B cells  CD3^-^/CD19^+^ | 10.83 ± 2.71 | 8.19 ± 8.44 | **2.96 ± 4.57***** | **4.73 ± 3.31***** | **7.28 ± 4.49*** |
| T cells  CD3^+^/CD56^-^ | 67.29 ± 7.41 | 64.93 ± 12.71 | 72.76 ± 13.06 | 50.72 ± 15.2 | 59 ± 10.84 |
| T helper cells  CD3^+^/CD4^+^ | 48.82 ± 9.24 | 39.21 ± 14.25 | 44.24 ± 9.96 | **29.43 ± 11.07***** | **31.69 ± 6.29***** |
| Cytotoxic T cells  CD3^+^/CD8^+^ | 10.83 ± 3.81 | 18.35 ± 10.16 | 18.67 ± 7.67 | 20.97 ± 9.32 | **22.44 ± 8.44*** |
| Tregs  CD3^+^/CD4^+^  CD25^+^/FoxP3^+^ | 9.92 ± 4.19 | 7.71 ± 2.15 | **5.93 ± 3.02**** | 9.25 ± 5.96 | 6.13 ± 0.98 |
| NKT cells  CD3^+^/CD56^+^ | 2.46 ± 1.95 | 6.07 ± 4.52 | 8.18 ± 5.25 | 7.33 ± 4.77 | 9.42 ± 7.78 |

Composition of lymphocyte subpopulations in % (mean value ± standard deviation) in healthy controls (Ctrl, n=22) and recurrent SCCHN patients (n=9) before (t0), after application of 20 to 30 Gy (t1), 3 months (t2) after RCT and at time of locoregional recurrence (t5, 3-15 months after t0). Significantly different values (ctrl vs. tx) are indicated in bold with an asterisk (*p ≤ 0.05; **p ≤ 0.01; ***p ≤ 0.001).

Supplementary Table 5: Significantly different values in lymphocyte subsets in the course of treatment (t0 vs. tx) in recurrent SCCHN patients

| % | t0 | t1 | t2 | t5 |
| --- | --- | --- | --- | --- |
| B cells  CD3^-^/CD19^+^ | 8.19 ± 8.44 | **2.96 ± 4.57*** | **4.73 ± 3.31*** | 7.28 ± 4.49 |
| T cells  CD3^+^/CD56^-^ | 64.93 ± 12.71 | 72.76 ± 13.06 | 50.72 ± 15.2 | 59 ± 10.84 |
| T helper cells  CD3^+^/CD4^+^ | 39.21 ± 14.25 | 44.24 ± 9.96 | 29.43 ± 11.07 | 31.69 ± 6.29 |
| Cytotoxic T cells  CD3^+^/CD8^+^ | 18.35 ± 10.16 | 18.67 ± 7.67 | 20.97 ± 9.32 | 22.44 ± 8.44 |
| Tregs  CD3^+^/CD4^+^  CD25^+^/FoxP3^+^ | 7.71 ± 2.15 | 5.93 ± 3.02 | 9.25 ± 5.96 | 6.13 ± 0.98 |
| NKT cells  CD3^+^/CD56^+^ | 6.07 ± 4.52 | **8.18 ± 5.25*** | 7.33 ± 4.77 | 9.42 ± 7.78 |

Composition of lymphocyte subpopulations in % (mean value ± standard deviation) in recurrent SCCHN patients (n=9) before (t0), after application of 20 to 30 Gy (t1), 3 months (t2) after RCT and at time of locoregional recurrence (t5, 3-15 months after t0). Significantly different values (t0 vs. tx) are indicated in bold with an asterisk (*p ≤ 0.05; **p ≤ 0.01; ***p ≤ 0.001).

Supplementary Table 6: Significantly different values in NK cell subpopulations in controls (Ctrl) vs. non-recurrent SCCHN patients

| % | Ctrl | t0 | t1 | t2 | t3 |
| --- | --- | --- | --- | --- | --- |
| NKT/NK cells  CD56^+^/CD69^+^ | 1.95 ± 2.64 | 1.95 ± 0.94 | 3.83 ± 2.14 | **3.65 ± 1.82*** | 3.13 ± 1.45 |
| NK cells  CD3^-^/CD56^+^ | 11.7 ± 5.15 | 11.1 ± 7.16 | 12.65 ± 7.03 | 15.83 ± 8.58 | 15.79 ± 7.8 |
| NK cells  CD3^-^/CD94^+^ | 7.42 ± 3.71 | 7.61 ± 3.85 | 9.86 ± 5.27 | **12.41 ± 6.5*** | **12.32 ± 5.07**** |
| NK cells  CD3^-^/NKG2D^+^ | 13.14 ± 5.05 | 10.64 ± 5.18 | 14.26 ± 8.33 | 14.15 ± 9.07 | 17.31 ± 7.94 |
| NK cells  CD3^-^/NKp30^+^ | 8.86 ± 4.75 | 7.95 ± 4.82 | 9.42 ± 5.55 | 13.39 ± 8.15 | **14.83 ± 8.66*** |
| NK cells  CD3^-^/NKp46^+^ | 11.97 ± 5.12 | 10.13 ± 4.87 | 12.16 ± 6.68 | 16.77 ± 9.23 | 16.94 ± 8.36 |

Composition of NK cell subpopulations in % (mean value ± standard deviation) in healthy controls (Ctrl, n=22) and non-recurrent SCCHN patients (n=23) before (t0), after application of 20 to 30 Gy (t1), 3 months (t2) and 6 months (t3) after RCT. Significantly different values (ctrl vs. tx) are indicated in bold with an asterisk (*p ≤ 0.05; **p ≤ 0.01; ***p ≤ 0.001).

Supplementary Table 7: Significantly different values in NK cell subpopulations in the course of treatment (t0 vs. tx) in non-recurrent SCCHN patients

| % | t0 | t1 | t2 | t3 |
| --- | --- | --- | --- | --- |
| NKT/NK cells  CD56^+^/CD69^+^ | 1.95 ± 0.94 | **3.83 ± 2.14*** | **3.65 ± 1.82**** | **3.13 ± 1.45**** |
| NK cells  CD3^-^/CD56^+^ | 11.1 ± 7.16 | 12.65 ± 7.03 | **15.83 ± 8.58**** | **15.79 ± 7.8**** |
| NK cells  CD3^-^/CD94+ | 7.61 ± 3.85 | 9.86 ± 5.27 | **12.41 ± 6.5**** | **12.32 ± 5.07***** |
| NK cells  CD3^-^/NKG2D^+^ | 10.64 ± 5.18 | **14.26 ± 8.33*** | **14.15 ± 9.07***** | **17.31 ± 7.94***** |
| NK cells  CD3^-^/NKp30^+^ | 7.95 ± 4.82 | 9.42 ± 5.55 | **13.39 ± 8.15***** | **14.83 ± 8.66***** |
| NK cells  CD3^-^/NKp46^+^ | 10.13 ± 4.87 | 12.16 ± 6.68 | **16.77 ± 9.23**** | **16.94 ± 8.36***** |

Composition of NK cell subpopulations in % (mean value ± standard deviation) in non-recurrent SCCHN patients (n=23) before (t0), after application of 20 to 30 Gy (t1), 3 months (t2) and 6 months (t3) after RCT. Significantly different values (t0 vs. tx) are indicated in bold with an asterisk (*p ≤ 0.05; **p ≤ 0.01; ***p ≤ 0.001).

**Supplementary Table 8:** Significantly different values in NK cell subpopulations in controls (ctrl) vs. recurrent SCCHN patients

| % | Ctrl | t0 | t1 | t2 | t5 |
| --- | --- | --- | --- | --- | --- |
| NKT/NK cells  CD56^+^/CD69^+^ | 1.95 ± 2.64 | 2.73 ± 1.73 | 3.65 ± 2.19 | 2.67 ± 0.6 | 3.05 ± 2 |
| NK cells  CD3^-^/CD56^+^ | 11.7 ± 5.15 | 7.91 ± 4.94 | 10.78 ± 7.2 | 11.28 ± 6.47 | 9.81 ± 6.53 |
| NK cells  CD3^-^/CD94^+^ | 7.42 ± 3.71 | 6.74 ± 4.23 | 8.97 ± 6.55 | 10.42 ± 6.88 | 9.74 ± 7.05 |
| NK cells  CD3^-^/NKG2D^+^ | 13.14 ± 5.05 | **7.98 ± 3.94*** | 9.8 ± 5.84 | 13.21 ± 8.1 | 11.45 ± 6.7 |
| NK cells  CD3^-^/NKp30^+^ | 8.86 ± 4.75 | 6.26 ± 4.74 | 9.25 ± 7.97 | 11.79 ± 7.45 | 5.88 ± 4.07 |
| NK cells  CD3^-^/NKp46^+^ | 11.97 ± 5.12 | **7.01 ± 5.47*** | 9.92 ± 8.38 | 10.52 ± 8.51 | 10.43 ± 7.19 |

Composition of NK cell subpopulations in % (mean value ± standard deviation) in healthy controls (Ctrl, n=22) and recurrent SCCHN patients (n=9) before (t0), after application of 20 to 30 Gy (t1), 3 months (t2) after RCT and at time of locoregional recurrence (t5, 3-15 months after t0). Significantly different values (ctrl vs. tx) are indicated in bold with an asterisk (*p ≤ 0.05; **p ≤ 0.01; ***p ≤ 0.001).

**Supplementary Table 9:** Significantly different values in NK cell subpopulations in the course of treatment (t0 vs. tx) in recurrent SCCHN patients

| % | t0 | t1 | t2 | t5 |
| --- | --- | --- | --- | --- |
| NKT/NK cells  CD56^+^/CD69^+^ | 2.73 ± 1.73 | 3.65 ± 2.19 | 2.67 ± 0.6 | 3.05 ± 2 |
| NK cells  CD3^-^/CD56^+^ | 7.91 ± 4.94 | 10.78 ± 7.2 | 11.28 ± 6.47 | 9.81 ± 6.53 |
| NK cells  CD3^-^/CD94^+^ | 6.74 ± 4.23 | 8.97 ± 6.55 | 10.42 ± 6.88 | **9.74 ± 7.05*** |
| NK cells  CD3^-^/NKG2D^+^ | 7.98 ± 3.94 | 9.8 ± 5.84 | 13.21 ± 8.1 | **11.45 ± 6.7*** |
| NK cells  CD3^-^/NKp30^+^ | 6.26 ± 4.74 | 9.25 ± 7.97 | 11.79 ± 7.45 | **5.88 ± 4.07*** |
| NK cells  CD3^-^/NKp46^+^ | 7.01 ± 5.47 | 9.92 ± 8.38 | 10.52 ± 8.51 | **10.43 ± 7.19*** |

Composition of NK cell subpopulations in % (mean value ± standard deviation) in recurrent SCCHN patients (n=9) before (t0), after application of 20 to 30 Gy (t1), 3 months (t2) after RCT and at time of locoregional recurrence (t5, 3-15 months after t0). Significantly different values (t0 vs. tx) are indicated in bold with an asterisk (*p ≤ 0.05; **p ≤ 0.01; ***p ≤ 0.001).
